# Mid-term shoulder functional and quality of life outcomes after shoulder replacement in obese patients

**DOI:** 10.1186/s40064-016-3624-0

**Published:** 2016-11-08

**Authors:** Heather K. Vincent, Aimee M. Struk, Austin Reed, Thomas W. Wright

**Affiliations:** 1 Department of Orthopedics and Rehabilitation, Division of Research, UF Orthopaedics and Sports Medicine Institute (OSMI), PO Box 112727, Gainesville, FL 32611 USA; 2 Department of Orthopaedics and Rehabilitation, University of Florida, 3450 Hull Road, 3rd Floor, Gainesville, FL 32608 USA; 3 Orthopaedics and Sports Medicine Institute, University of Florida, 3450 Hull Road, Gainesville, FL 32611 USA

**Keywords:** Shoulder, Obese, Physical function, Outcomes, Range of motion

## Abstract

**Background:**

Shoulder pain and loss of function are directly associated with obesity.

**Questions/purposes:**

We hypothesized that significant interactions would exist between total shoulder arthroplasty (TSA) and reverse total shoulder arthroplasty (RSA) and obesity status on functional and quality of life (QOL) outcomes over the long term. Clinical and QOL outcomes (American Shoulder and Elbow Surgeons Evaluation form, Shoulder Pain and Disability Index, University of California at Los Angeles Shoulder Rating scale, Medical Outcomes Short Form 12 (SF-12), range of motion (ROM), and strength) were longitudinally compared in patients with low and high body mass index (BMI) after a TSA or a RSA. Prospectively collected data of patients with a TSA or RSA were reviewed (N = 310). Preoperative, 2-year, and final follow-up visits were included (range 3–17 years; mean 5.0 ± 2.5 years). Patient data were stratified for analysis using BMI.

**Results:**

Morbidly obese patients had worse preoperative functional scores and QOL compared to the other groups. There were no significant interactions of BMI group by surgery type for any of the outcome variables except for active external rotation ROM. Morbidly obese patients attained lower SF-12 scores compared to the remaining groups at each time point.

**Conclusions:**

Both TSA and RSA can be expected to impart positive functional outcomes in patients irrespective of BMI. Morbidly obese patients do not attain the same gains in Medical Outcomes SF-12 scores as the non-morbidly obese patients. The lower improvements in active external ROM may be due to morphological limitations of excessive adiposity.

**Level of evidence:**

This is a level II study.

## Background

Shoulder pain and loss of function are directly associated with obesity (Kane et al. 2010). High body mass index (BMI) increases incidence of rotator cuff tendinitis (Wendelboe et al. 2004), arthropathy, rotator cuff tear size (Gumina et al. 2014), and the need for elective shoulder replacement (Bostman 1994). The annual volume of total shoulder arthroplasty (TSA) and reverse total shoulder arthroplasty (RSA) has increased 2.5 fold in the U.S. from 2000 to 2008 (Beck et al. 2013; Bostman 1994). The percentage of overweight and obese patients undergoing TSA or RSA ranges from 19 to 75% (Gupta et al. 2014) depending on the cohort (Gupta et al. 2014; Werner et al. 2015b). TSAs and RSAs are often indicated for treatment of osteoarthritis of the shoulder. RSAs are typically indicated for rotator cuff tear arthropathy, massive rotator cuff tears, proximal humerus fractures, revision arthroplasty, and glenohumeral osteoarthritis in the context of irreparable rotator cuff tears (Saltzman et al. 2014) and the indications for use are continuing to expand (Urch et al. 2016).

The relationship between BMI and shoulder replacement outcome is complex. High BMI increases the surgical complexity and risk for complications out to 6 months (Beck et al. 2013; Griffin et al. 2014; Gupta et al. 2014; Werner et al. 2015a, b), but may not impact 30-day complications (Jiang et al. 2016). Obesity does not necessarily inhibit gains in outcome scores and quality of life (QOL) over the short-term (Li et al. 2013). Few studies have assessed obesity’s collective impact on pain, range of motion (ROM), or QOL after shoulder replacement (Li et al. 2013; Linberg et al. 2009; Pappou et al. 2014). Limited evidence suggests that obese patients can attain similar 2-year improvements in physical health as non-obese patients after TSA (Li et al. 2013) and can attain good surgical outcomes after RSA (Statz et al. 2016). What remains unclear is whether shoulder-specific outcomes, physical function, and patient-reported QOL are different beyond 2 years or longer across different BMI strata. This evidence gap is significant; comparative patient-centric metrics are not yet available to facilitate the shared decision-making process between patient and surgeon. An improved understanding of the mechanics of RSA and prostheses permitted the expansion of indications that are considered appropriate for this procedure (Urch et al. 2016), thereby increasing the likelihood that its use in patients with high BMI values becomes common. Moreover, as the indications for the use of RSA expand, it is not clear whether obesity differentially impacts these actual or patient-reported outcomes over a longer time frame.

Previous work has shown that morbidly obese patients have significantly worse functional outcomes than non-obese counterparts after rehabilitation for lower body elective procedures such as hip and knee arthroplasty (Vincent and Vincent 2008; Vincent et al. 2007). It is still unclear whether mid-term TSA and RSA actual and patient-reported outcomes are different based on BMI value, which precludes establishment of expected mid-term elective shoulder replacement outcomes and risk analysis. It is possible that despite the different indication for TSA and RSA and different initial functional ability, obese patients may report similar perceived improvements in QOL as non-obese patients. Clarifying the relationship of BMI strata to outcomes after TSA and RSA will provide the first complete patient experience that can be used to establish expectations for improvement in patient subgroups that may benefit from additional interventions. Comparative evidence of sustained outcomes is essential as health care moves toward a pay-for-performance and value-based system (Li et al. 2013). Thus, our study first compared surgery-specific and general health QOL outcomes between non-obese, obese, and morbidly obese patients with a TSA or RSA. We hypothesized that significant interactions would exist between the surgery type and obesity status on functional and QOL outcomes over the long term. Although all patients would experience improved functional outcomes and QOL, we anticipated that morbidly obese patients would report lower QOL and physical function gains than non-obese patients after TSA and RSA.

## Methods

### Study design

A retrospective review was conducted of prospectively collected data on patients who underwent TSA or RSA between January of 1992 and May of 2012 at the University of Florida. Both TSA and RSA were included in this analysis as RSA represents approximately one-third of the nationally reported shoulder replacement surgeries in the U.S. (Westermann et al. 2015). Diagnoses included osteoarthritis, acute fracture, rotator cuff tear, and rotator cuff arthropathy, and other (osteonecrosis, avascular necrosis, and synovial chondromatosis).

Of the enrolled patients, 310 had complete follow-up data for our research questions. Patients underwent TSA or RSA and had a minimum 2-year follow up and a final mid-term mean follow up of 5.0 ± 2.5 years (range 3–17 years). Age ranged from 21 to 95 years. Study documents and procedures were approved by the University of Florida Institutional Review Board and procedures conformed to the guidelines set by the Declaration of Helsinki for the treatment of human subjects.

Patients were stratified into groups based on BMI values: non-obese (BMI < 30 kg/m^2^), obese (30–39.9 kg/m^2^), and morbidly obese (≥40 kg/m^2^) for statistical analysis (Kane et al. 2010). Demographics (age, height, weight, BMI, and ethnicity/race), surgery type (TSA or RSA), comorbidities, perioperative analgesic use, and tobacco use were collected preoperatively.

### Patient-reported outcome measures

Surveys were administered to each participant on subjective shoulder-specific functional and pain outcomes, perceived physical activity, and general health.

#### American Shoulder and Elbow Surgeons Evaluation form (ASES)

The ASES permits the patient to self-evaluate pain, shoulder instability, and activities of daily living (Richards et al. 1994). Eleven items are used to generate a score (pain has one item, function ten items). The physician-assessment section documents ROM, physical signs, strength, and stability. The final score is derived from the pain and cumulative activities of daily living score (Richards et al. 1994). A minimal clinically important difference is 6.4 points (Roy et al. 2009).

#### Shoulder Pain and Disability Index (SPADI)

The SPADI assesses shoulder pain and disability in the outpatient setting (Breckenridge and McAuley 2011), with 13 items categorized into two subscales: pain (five items) and disability (eight items) (Roach et al. 1991). This instrument has high internal consistency with Chronbach’s α values ranging from 0.86 to 0.95 for the subscales. The SPADI responds to change over time in various patient populations, and can discriminate between patients with improving and deteriorating conditions (Beaton and Richards 1996; Roy et al. 2009; Williams et al. 1995). A minimal clinically important difference is 8 points (Roy et al. 2009).

#### University of California at Los Angeles Shoulder Rating scale (UCLA)

This scale has separate domain areas: pain, function, strength of forward flexion, active forward flexion, and overall patient satisfaction (Amstutz et al. 1981). The scale is weighted by pain, and function accounts for 20 points. The other domains account for 15 points, giving a total score of 35 points (<27 is poor and fair and >27 is good to excellent). This has been used in patients with shoulder replacement due to osteoarthritis, rotator cuff disease, and instability (Kirkley et al. 2003).

#### Constant score

This score is comprised of subjective assessments relating to pain, activities of daily living, and objective measures of shoulder motion and strength. The maximum score is 100 points, with higher scores representing less pain and better shoulder function (Constant et al. 2008). Construct validity is 6 = .673, and the Constant score correlates moderately well with the UCLA score (r = 0.673) (Oh et al. 2009).

#### Medical Outcomes Short Form 12 (SF-12)

The SF-12 is a standardized, validated health related QOL questionnaire comprised of 12 items of the Medical Outcomes Short Form 36 original instrument (Ware et al. 1996). The SF-12 summary score (PCS-12 and MCS-12) ranges from 0 to 100 points. The SF-12 has a high test–retest reliability (r = 0.89) and has been validated for use in independently living older adults with chronic illnesses (Resnick and Nahm 2001).

### Radiological outcomes and adverse events

Development of radiolucency lines during the follow-up (specifically within the glenoid) and scapular notching was identified by the surgeon in the study (Choi et al. 2013). Adverse events included infections, bone graft failure, loosening, dislocation, rotator cuff failure, wound complications, fracture, and pain and stiffness. The number of revisions in each surgery category was documented. The criteria for revisions included the following: infections, loosening or instability, dislocation, bone graft failure, and rotator cuff repair failure. If revisions occurred, the follow-up time was determined from the initial procedure.

### Objective functional measures

Passive and active shoulder ROM was assessed by an experienced athletic trainer using a goniometer. Active external rotation and active elevation measures were performed by the patient. Each participant lifted progressively higher weights in increments of 2.2 kg during a lateral straight arm raise to estimate strength. The strength value was defined as the maximal weight the patient could lift with appropriate form once.

### Statistical analyses

Data were analyzed using the Statistical Package for the Social Sciences (SPSS version 24.0, IBM Corp; Chicago, IL). Patient characteristics were compared between the three BMI strata (non-obese, overweight, and obese) using one-way analysis of variance (ANOVA) and a Scheffé post hoc analysis for continuous variables and by Kruskal–Wallis tests for categorical variables. Chi square tests were used to determine whether distributions of the shoulder pain mechanisms, radiolucency lines, and scapular notching were different among the groups. To address the purposes of the study, generalized linear models were used, where the dependent variables were study outcomes (ASES, SPADI, UCLA, Constant scores, SF-12, range of motion and strength) and the predictors were obesity status (non-obese, obese, and morbidly obese) and surgical type (RSA or TSA). Covariates were diagnosis and age. Significance was established at p < .05 a priori for all tests.

## Results

Table 1 shows patient characteristics. The morbidly obese group had a higher prevalence of women and Diabetes Mellitus than the other groups (p < .05). The obese group had a greater proportion of patients with hypertension and those who took analgesics for pain than the remaining groups (p < .05). A total of 56.9% of patients underwent a TSA and 43.1% underwent a RSA. There was a significant difference in the distributions of shoulder pain mechanisms among the BMI groups (χ^2^ = 52.417; p < .0001).

**Table 1:** Participant characteristics of the study groups

	Non-obese (<25 kg/m^2^) n = 167	Obese (30–39.9 kg/m^2^) n = 121	Morbidly obese (≥40 kg/m^2^) n = 22	p (sig)
Age (years)	69.1 ± 10.4	69.3 ± 8.5	66.9 ± 5.2	0.124
Weight (kg)	73.6 ± 12.4	94.1 ± 13.9	126.2 ± 19.5	0.0001
BMI (kg/m^2^)	26.1 ± 2.5	34.1 ± 2.9	47.3 ± 5.6	0.0001
Sex (%)				
Male	50	49.5	27	
Female	50	50.5	73	0.002
Ethnicity (%)				
Caucasian	95.7	95.1	93.7	
African American	2	1.2	6.3	
Hispanic	2.3	0.9	0	
Asian	0	0.9	0	
Other	0	1.9	0	0.467
Comorbidities (%)				
Hypertension	23	38.5	30.2	0.0001
Heart disease	9.3	9.8	4.8	0.436
Diabetes mellitus	9.3	10.2	23.8	0.002
Tobacco use (%)	4.1	3.1	0	0.692
Analgesic use (%)	25.2	36	27	0.004
Mechanism of shoulder pain (%)				
Acute fracture	4.1	1.8	0	
Osteoarthritis	43.7	52.9	63.5	
Rotator cuff tear	1.3	3.7	4.8	
Rotator cuff arthropathy	35.7	30.8	20.6	
Other	15.2	10.8	11.1	0.0001

Values are mean ± SD or percent of the group

*BMI* body mass index

Adverse events that did and did not require revision are shown in Table 2 for the surgery types and BMI groups. The numbers of radiolucent lines (humeral and glenoid) and scapular notching at follow-ups are shown in Table 3. The distributions of the radiolucency lines (glenoid and humeral) and scapular notching were not different among the BMI groups. The revision procedures that were performed included the following: removal and replacement of long-stem humeral implant, revision to hemiarthroplasty, removal of loose glenoid and revision, conversion of TSA to RSA, reduction and revision, revision to an antibiotic spacer, and bone grafting for injury after a fall.

**Table 2:** Adverse events (AE) in patients from each body mass index (BMI) stratum

	Non-obese n = 167	Obese n = 121	Morbidly obese
*AE not requiring revision*			
TSA (five patients, six AE)			
Loosening	1	0	0
RCR failure	2	0	0
Wound complication	0	1	0
General pain and stiffness	2	0	0
RSA (nine patients, ten AE)			
Fracture	5	4	0
Dislocation	1	0	0
*Revision required* <*2* *years*			
TSA (seven patients, seven AE)			
Infection	0	1	0
Loosening	1	0	1
RCR failure	4	0	0
RSA (five patients, seven AE)			
Infection	2	0	0
Loosening	1	0	0
Dislocation/unstable	4^a^	0	0
*Revision required* >*2* *years*			
TSA (13 patients, 17 AE)			
Infection	3	1	0
Loosening	5	1	0
Dislocation/unstable	1	0	0
Fracture	1	1	0
Bone graft failure	0	0	0
RCR failure	3	1	0
RSA (four patients, seven AE)			
Infection	0	1	0
Loosening	2	1	0
Fracture	0	1	0
Bone graft failure	1	0	0
RCR failure	1	0	0

*TSA* total shoulder arthroplasty, *RSA* reverse shoulder arthroplasty, *RCR* rotator cuff repair

^a^Three dislocations in the same patient

**Table 3:** Radiolucency lines and scapular notching by the final follow-up time point

	Non-obese n	Obese	Morbidly obese	p (sig)
Humeral radiolucent lines visible	7 (seven patients)	5 (five patients)	1 (one patient)	0.999
Glenoid radiolucent lines visible	97 (48)	76 (38)	10 (9)	0.376
Scapular notching	12 (11)	4 (4)	0 (0)	0.580

Values represent number and patient number (in parentheses)

Patient-reported functional outcomes are provided in Table 4. There were no significant interactions of obesity status by time for any outcome variable. There were no significant main effects of obesity or surgery type for any outcome. Improvements were maintained at the final follow-up.

**Table 4:** Patient-reported functional outcomes

	Non-obese (<25 kg/m^2^)	Obese (30–39.9 kg/m^2^)	Morbidly obese (≥40 kg/m^2^)	p (sig) obesity	p (sig) surgery type	p (sig) obesity * surgery interaction
ASES						
Preoperative	39.5 ± 16.6	36.7 ± 14.8	27.1 ± 10.6			
2 years	80.1 ± 18.0	81.4 ± 18.2	72.3 ± 16.6			
Follow-up	74.8 ± 20.5	78.9 ± 19.8	68.2 ± 23.4	0.114	0.661	0.313
SPADI						
Preoperative	65.5 ± 16.2	67.3 ± 13.5	68.9 ± 7.5			
2 years	20.6 ± 18.8	20.3 ± 21.2	32.1 ± 17.7			
Follow-up	25.9 ± 21.1	21.9 ± 19.3	33.4 ± 23.7	0.205	0.265	0.770
UCLA						
Preoperative	13.9 ± 4.3	13.8 ± 3.8	13.7 ± 0.6			
2 years	29.0 ± 5.2	29.3 ± 5.8	26.6 ± 6.8			
Follow-up	27.6 ± 6.1	28.7 ± 5.4	25.7 ± 7.0	0.766	0.540	0.373
Constant score (normalized)						
Preoperative	39. 5 ± 15.3	37.3 ± 12.5	32.1 ± 11.2			
2 years	73.7 ± 13.9	73.8 ± 14.3	62.3 ± 15.6			
Follow-up	70.0 ± 15.6	73.9 ± 13.7	63.6 ± 19.6	0.826	0.426	0.523

Values are expressed in points and are shown as mean ± SD. Significance is the interaction of time point by obesity status

*ASES* American Shoulder and Elbow Surgeons survey, *UCLA* University of California at Los Angeles Shoulder Rating scale, *SPADI* Shoulder Pain and Disability Index

Patient-reported QOL SF-12 scores are presented in Table 5. There were no significant interactions of obesity status by time for the SF-12 scores. However, there was a main effect for obesity for all SF-12 scores (p < 0.05). All BMI groups improved the SF-12 scores over time, but the morbidly obese group attained lower SF-12 scores compared to the remaining groups at each time point (p < .0001).

**Table 5:** Quality of life (QOL) represented by the Medical Outcomes Short Form 12 (SF-12) total score, physical component score (PCS), and mental component score (MCS)

	Non-obese (<25 kg/m^2^)	Obese (30–39.9 kg/m^2^)	Morbidly obese (≥40 kg/m^2^)	p (sig) obesity	p (sig) surgery type	p (sig) obesity * surgery interaction
SF-12 total						
Preoperative	32.6 ± 6.6	31.4 ± 6.4	24.7 ± 5.7			
2 years	37.6 ± 6.4	36.7 ± 6.9	32.1 ± 6.6			
Follow-up	36.3 ± 7.2	35.9 ± 7.3	30.8 ± 3.9	0.0001	0.714	0.541
SF-12 PCS						
Preoperative	12.9 ± 2.9	11.9 ± 2.9	10.0 ± 2.1			
2 years	15.4 ± 3.4	14.9 ± 3.5	12.3 ± 3.1			
Follow-up	14.8 ± 3.7	14.5 ± 3.6	11.2 ± 2.1	0.0001	0.477	0.910
SF-12 MCS						
Preoperative	19.6 ± 4.2	19.4 ± 4.4	14.6 ± 4.2			
2 years	22.2 ± 3.7	21.8 ± 3.7	19.7 ± 4.0			
Follow-up	21.5 ± 4.2	21.4 ± 4.3	19.6 ± 3.4	0.0001	0.926	0.508

Values are expressed in points and are shown in mean ± SD

Objective functional scores of active and passive ROM and shoulder strength are presented in Table 6. Only one significant obesity status by time interaction existed for active external rotation (p = .021), with main effects of both obesity and surgery type reaching significance.

**Table 6:** Shoulder motion and strength

	Non-obese (<25 kg/m^2^)	Obese (30–39.9 kg/m^2^)	Morbidly obese (≥40 kg/m^2^)	p (sig) obesity	p (sig) surgery type	p (sig) interaction
*Active motion*						
External rotation (°)						
Preoperative	8.9 ± 5.5	9.1 ± 5.8	11.6 ± 7.1			
2 years	10.7 ± 6.0	13.4 ± 8.1	13.4 ± 6.1			
Follow-up	10.9 ± 6.2	12.2 ± 6.4	12.9 ± 6.4	0.0001	0.0001	0.021
Elevation (°)						
Preoperative	85.1 ± 32.2	84.1 ± 29.8	79.1 ± 32.7			
2 years	120.1 ± 24.6	124.9 ± 24.6	118.3 ± 36.5			
Follow-up	118.6 ± 28.7	120.6 ± 27.8	115.9 ± 36.8	0.536	0.940	0.734
*Passive motion*						
External rotation (°)						
Preoperative	35.2 ± 23.5	28.3 ± 17.7	29.2 ± 18.6			
2 years	51.7 ± 17.3	51.1 ± 17.5	48.1 ± 18.7			
Follow-up	52.5 ± 18.3	49.1 ± 15.7	52.1 ± 15.1	0.051	0.204	0.171
Elevation						
Preoperative	118.1 ± 30.0	112.5 ± 31.9	106.1 ± 29.9			
2 years	140.2 ± 17.7	145.3 ± 14.9	142.7 ± 19.4			
Follow-up	138.2 ± 20.9	142.4 ± 17.5	145.4 ± 18.7	0.850	0.047	0.121
*Maximal weight able to lift*						
Weight lifted (kg)^a^						
Preoperative	0.7 ± 1.3	0.4 ± 1.1	0.0			
2 years	1.9 ± 2.0	2.2 ± 2.0	0.9 ± 1.4			
Follow-up	1.6 ± 1.8	2.2 ± 2.2	1.8 ± 2.6	0.301	0.780	0.301

Values are mean ± SD. Significance is the interaction of time point by obesity status

^a^Weight was lifted in shoulder abduction with a fully extended arm; weight increments used for testing were in 2.2 kg

## Discussion

The interactions between surgery type and obesity status on several mid-term shoulder-specific general outcomes were tested. The novel findings are (a) morbidly obese patients made significant improvements in functional and QOL outcomes over the mid-term; (b) there were no significant main effects for surgery type on ASES, SPADI, UCLA, and Constant scores; and (c) shoulder replacement adverse events and radiological outcomes were not different among the BMI groups. These findings demonstrate sustained benefit of both surgery types on clinically meaningful outcomes even in patients with high BMIs. The magnitude of improvement in QOL was less in the morbidly obese patients compared with the remaining groups by follow-up.

General health QOL and ROM/strength assessments were compared among non-obese, obese, and morbidly obese patients. Surgery benefits on outcomes were maintained past 2 years, suggesting that shoulder arthroplasty effectively relieves symptoms and improves function in patients across the BMI spectrum. Because morbidly obese patients had worse function and QOL before the surgery, they did not achieve the same absolute level of improvement as the other BMI groups on the ASES, SPADI, and UCLA scores. Improvements in these outcomes ranged from 48 to 65% in the morbidly obese group, and from 60 to 115% in the other groups. Despite the lesser gain in perceived function, morbidly obese groups achieved similar relative improvements in the SF-12 physical component score and over twice the improvement in the SF-12 mental component score than the other BMI groups. Few directly comparable data are available in obese patients. However, patient-reported QOL after RSA has been shown to reach comparable levels to that of healthy age-matched norms using the SF-36. Also, SF-36 domains have been shown to be higher than those reported by a normalized age-matched cohort after TSA (Gruson et al. 2010).

Recent studies measured shoulder replacement outcomes in obese patients. First, a case control study of obese and non-obese patients (N = 84) with RSA similarly found that obese patients did not achieve the same ASES improvement as non-obese controls after surgery (86 vs. 105%) (Pappou et al. 2014). Pain subscores improved similarly in the two patients groups (78–80%), but the greatest change occurred in the ASES function subscores in the non-obese and obese patients (142 vs. 89%). Second, a prospective study of patients enrolled in a shoulder registry (N = 76) collected preoperative and 2-year ASES and SF-36 scores, and visual analogue scales of pain and fatigue (Li et al. 2013). Patients were stratified into ‘‘normal’’ weight, overweight, and obese groups based on BMI. The ASES scores improved by 108 and 123% in the normal weight and obese patients, respectively. Improvements on the SF-36 physical and mental subscores were less in the obese patients than the normal weight patients, however. The obese patients experienced 2.1–12% improvements in the physical SF-36 subscores compared to the normal weight patients, who experienced improvements ranging from 11.3 to 40.2%. Third, a study of morbidly obese patients with TSA (N = 45 shoulders) found a 53.5% reduction in pain out to an average of 4.6 years of follow up (Linberg et al. 2009). These studies and ours suggest that obese patients may report improvements in shoulder-related function, but potentially less improvement in reported QOL.

Individuals with low and high BMI values can improve active elevation, external rotation, and internal rotation abduction and forward flexion after TSA and RSA (Constant et al. 2008; Gupta et al. 2014). We observed improvements in active (external rotation, active elevation) and passive (external rotation, elevation) ROM between morbidly obese, obese, and non-obese patients. Shoulder raise strength gains were made across all groups, with continued strength gain in the morbidly obese group. Obesity reduces upper body muscle strength and endurance (Cavuoto and Nussbaum 2014), compromises shoulder ROM as much as 38.9% for actions like shoulder abduction (Park et al. 2010), and increases upward scapular rotation during movement (Gupta et al. 2013). The combination of low muscle strength and shoulder ROM negatively impacts the ability to perform daily activities. Thus, improvements in ROM and strength in obese patients after surgery might translate to sustained performance of activities of daily living over time (Maier et al. 2014).

Case–control data comparing outcomes between TSA and RSA revealed that ASES scores, pain severity, elevation, abduction, and internal rotation improved similarly after the two procedures (Kiet et al. 2015). After RSA and TSA, a significant proportion of patients continue to participate in medium or high-demand activities (84 and 89%, respectively), but there are specific activities that people with RSA are unable to do well (Ware et al. 1996). Morbidly obese patients may experience unique difficulties with shoulder movements that are not captured with current instruments. Development of task inventories that may be relevant to an obese person may provide researchers and clinicians with better insight into the impact of the surgery on shoulder function. Surgeons and patients would benefit from discussing functional and QOL goals to determine which procedure would yield the best results (Schwarzkopf et al. 2013). Here, the morbidly obese group’s pain may be more related to degenerative disease and less to rotator cuff issues, whereas the non-obese group’s pain may be more linked with rotator cuff arthropathy and acute fracture. The fact that there were no differences in adverse events and radiological outcomes in the three patient groups indicates that obesity does not compromise the success of shoulder replacement. Surgical possibilities are open to both procedures, even in morbidly obese patients.

To our knowledge, this is the first study to examine the mid-term changes in function and patient-reported QOL after two shoulder replacement surgery types. Some limitations and strengths deserve comment. First, the patient subgroup sizes are different, with the morbidly obese subgroup being the smallest size. Based on clinical tracking of patient population demographics, we believe that this distribution represents the actual proportions of patients seen in this tertiary care institution. This study collected a battery of standardized assessments, most of which were self-report surveys. These surveys were joint-specific and general, providing a more comprehensive assessment of the patient experience. The same investigators administered the surveys and performed functional testing in all patients, minimizing interrater error.

## Conclusions

Shoulder replacement procedures improve function and QOL outcomes in patients across the BMI spectrum out to an average of 5 years. The interactions of obesity status and surgery type for key outcomes were not different in the non-obese, obese, and morbidly obese patients. Adverse event frequencies and radiological outcomes were not higher in obese and morbidly obese patients. Morbidly obese patients can achieve meaningful shoulder outcomes and QOL without elevated risk for adverse events or worse radiological outcomes over the mid-term.

## Abbreviations

BMI: body mass index
TSA: total shoulder arthroplasty
RSA: reverse total shoulder arthroplasty
QOL: quality of life
ROM: range of motion
ASES: American Shoulder and Elbow Surgeons Evaluation form
SPADI: Shoulder Pain and Disability Index
UCLA: University of California at Los Angeles Shoulder Rating scale
SF-12: Medical Outcomes Short Form 12
ANOVA: analysis of variance

## References

[CR1] Amstutz HC, Sew Hoy AL, Clarke IC (1981). UCLA anatomic total shoulder arthroplasty. Clin Orthop.

[CR2] Beaton DE, Richards RR (1996). Measuring function of the shoulder. A cross-sectional comparison of five questionnaires. J Bone Joint Surg.

[CR3] Beck JD, Irgit KS, Andreychik CM, Maloney PJ, Tang X, Harter GD (2013). Reverse total shoulder arthroplasty in obese patients. J Hand Surg.

[CR4] Bostman OM (1994). Prevalence of obesity among patients admitted for elective orthopaedic surgery. Int J Obes Relat Metab Disord J Int Assoc Study Obes.

[CR5] Breckenridge JD, McAuley JH (2011). Shoulder pain and disability index (SPADI). J Physiother.

[CR6] Cavuoto LA, Nussbaum MA (2014). The influences of obesity and age on functional performance during intermittent upper extremity tasks. J Occup Environ Hyg.

[CR7] Choi T, Horodyski M, Struk AM, Sahajpal DT, Wright TW (2013). Incidence of early radiolucent lines after glenoid component insertion for total shoulder arthroplasty: a radiographic study comparing pressurized and unpressurized cementing techniques. J Shoulder Elbow Surg.

[CR8] Constant CR, Gerber C, Emery RJH, Søjbjerg JO, Gohlke F, Boileau P (2008). A review of the Constant score: modifications and guidelines for its use. J Shoulder Elbow Surg.

[CR9] Griffin JW, Novicoff WM, Browne JA, Brockmeier SF (2014). Morbid obesity in total shoulder arthroplasty: risk, outcomes, and cost analysis. J Shoulder Elbow Surg.

[CR10] Gruson KI, Pillai G, Vanadurongwan B, Parsons BO, Flatow EL (2010). Early clinical results following staged bilateral primary total shoulder arthroplasty. J Shoulder Elbow Surg.

[CR11] Gumina S, Candela V, Passaretti D, Latino G, Venditto T, Mariani L, Santilli V (2014). The association between body fat and rotator cuff tear: the influence on rotator cuff tear sizes. J Shoulder Elbow Surg.

[CR12] Gupta M, Dashottar A, Borstad JD (2013). Scapula kinematics differ by body mass index. J Appl Biomech.

[CR13] Gupta AK, Chalmers PN, Rahman Z, Bruce B, Harris JD, McCormick F, Abrams GD, Nicholson GP (2014). Reverse total shoulder arthroplasty in patients of varying body mass index. J Shoulder Elbow Surg.

[CR14] Jiang JJ, Somogyi JR, Patel PB, Koh JL, Dirschl DR, Shi LL (2016). Obesity is not associated with increased short-term complications after primary total shoulder arthroplasty. Clin Orthop Relat Res.

[CR15] Kane S, Conus S, Haltom D, Hirshorn K, Pak Y, Vigdorchik J (2010). A shoulder health survey: correlating behaviors and comorbidities with shoulder problems. Sports Health Multidiscip Approach.

[CR16] Kiet TK, Feeley BT, Naimark M, Gajiu T, Hall SL, Chung TT, Ma CB (2015). Outcomes after shoulder replacement: comparison between reverse and anatomic total shoulder arthroplasty. J Shoulder Elbow Surg.

[CR17] Kirkley A, Griffin S, Dainty K (2003). Scoring systems for the functional assessment of the shoulder. Arthrosc J Arthrosc Relat Surg.

[CR18] Li X, Williams PN, Nguyen JT, Craig EV, Warren RF, Gulotta LV (2013). Functional outcomes after total shoulder arthroplasty in obese patients. J Bone Joint Surg.

[CR19] Linberg CJ, Sperling JW, Schleck CD, Cofield RH (2009). Shoulder arthroplasty in morbidly obese patients. J Shoulder Elbow Surg.

[CR20] Maier MW, Caspers M, Zeifang F, Dreher T, Klotz MC, Wolf SI, Kasten P (2014). How does reverse shoulder replacement change the range of motion in activities of daily living in patients with cuff tear arthropathy? A prospective optical 3D motion analysis study. Arch Orthop Trauma Surg.

[CR21] Oh JH, Jo KH, Kim WS, Gong HS, Han SG, Kim YH (2009). Comparative evaluation of the measurement properties of various shoulder outcome instruments. Am J Sports Med.

[CR22] Pappou I, Virani NA, Clark R, Cottrell BJ, Frankle MA (2014). Outcomes and costs of reverse shoulder arthroplasty in the morbidly obese. J Bone Joint Surg.

[CR23] Park W, Ramachandran J, Weisman P, Jung ES (2010). Obesity effect on male active joint range of motion. Ergonomics.

[CR24] Resnick B, Nahm ES (2001). Reliability and validity testing of the revised 12-item Short-Form Health Survey in older adults. J Nurs Meas.

[CR25] Richards RR, An K-N, Bigliani LU, Friedman RJ, Gartsman GM, Gristina AG, Iannotti JP, Mow VC, Sidles JA, Zuckerman JD (1994). A standardized method for the assessment of shoulder function. J Shoulder Elbow Surg.

[CR26] Roach KE, Budiman-Mak E, Songsiridej N, Lertratanakul Y (1991). Development of a shoulder pain and disability index. Arthritis Care Res Off J Arthritis Health Prof Assoc.

[CR27] Roy J-S, MacDermid JC, Woodhouse LJ (2009). Measuring shoulder function: a systematic review of four questionnaires. Arthritis Care Res.

[CR28] Saltzman BM, Chalmers PN, Gupta AK, Romeo AA, Nicholson GP (2014). Complication rates comparing primary with revision reverse total shoulder arthroplasty. J Shoulder Elbow Surg.

[CR29] Schwarzkopf R, Lerebours F, Walsh M, Zuckerman JD, Loebenberg MI (2013). Shoulder arthroplasty expected outcomes: surgeons’ opinion survey. Bull Hosp Joint Dis.

[CR30] Statz JM, Wagner ER, Houdek MT, Cofield RH, Sanchez-Sotelo J, Elhassan BT, Sperling JW (2016). Outcomes of primary reverse shoulder arthroplasty in patients with morbid obesity. J Shoulder Elbow Surg.

[CR31] Urch E, Dines JS, Dines DM (2016). Emerging indications for reverse shoulder arthroplasty. Instr Course Lect.

[CR32] Vincent HK, Vincent KR (2008). Obesity and inpatient rehabilitation outcomes following knee arthroplasty: a multicenter study. Obes Silver Spring Md.

[CR33] Vincent HK, Weng JP, Vincent KR (2007). Effect of obesity on inpatient rehabilitation outcomes after total hip arthroplasty. Obes Silver Spring Md.

[CR34] Ware J, Kosinski M, Keller SD (1996). A 12-Item Short-Form Health Survey: construction of scales and preliminary tests of reliability and validity. Med Care.

[CR35] Wendelboe AM, Hegmann KT, Gren LH, Alder SC, White GL, Lyon JL (2004). Associations between body-mass index and surgery for rotator cuff tendinitis. J Bone Joint Surg Am.

[CR36] Werner BC, Burrus MT, Browne JA, Brockmeier SF (2015). Superobesity (body mass index > 50 kg/m2) and complications after total shoulder arthroplasty: an incremental effect of increasing body mass index. J Shoulder Elbow Surg.

[CR37] Werner BC, Griffin JW, Yang S, Brockmeier SF, Gwathmey FW (2015). Obesity is associated with increased postoperative complications after operative management of proximal humerus fractures. J Shoulder Elbow Surg.

[CR38] Westermann RW, Pugely AJ, Martin CT, Gao Y, Wolf BR, Hettrich CM (2015). Reverse shoulder arthroplasty in the United states: a comparison of national volume, patient demographics, complications, and surgical indications. Iowa Orthop J.

[CR39] Williams JW, Holleman DR, Simel DL (1995). Measuring shoulder function with the shoulder pain and disability index. J Rheumatol.

